# Knowledge and practice of implant-retained restorations among dental students in Saudi Arabia

**DOI:** 10.12669/pjms.314.7384

**Published:** 2015

**Authors:** Fahim Vohra, Altaf Hussain Shah, Mohammad Sohail Zafar, Zaheer Kola

**Affiliations:** 1 Dr. Fahim Vohra, Associate Professor, Department of Prosthetic Dental Sciences, College of Dentistry, King Saud University, Riyadh, Saudi Arabia; 2 Dr. Altaf Hussain Shah, Department of Preventive Dental Sciences, College of Dentistry, Prince Salman Bin Abdulaziz University, Al-Kharj, Saudi Arabia; 3 Dr. Mohammad Sohail Zafar, Assistant Professor, Department of Restorative Dentistry, College of Dentistry, Taibah University, Al-Madinah Al-Munawarah, Saudi Arabia; 4 Dr. Zaheer Kola, Department of Prosthetic Dental Sciences, College of Dentistry, Prince Salman Bin Abdulaziz University, Al-Kharj, Saudi Arabia

**Keywords:** Knowledge, Practice, Student, Implant restoration

## Abstract

**Objectives:**

The aim of the study was to assess the knowledge and practice of implant retained restorations (IRR) among senior dental students in Saudi Arabia.

**Methods:**

Four hundred questionnaires were distributed among senior dental students of five dental schools in Saudi Arabia. Student’s knowledge was assessed regarding which implant restoration [cement retained restoration (CRR) or screw retained restoration (SRR)] better provides the desired clinical properties. Students’ practice of IRR, perception of their knowledge and need for further education related to IRR were also assessed. Descriptive statistics and chi-square test were employed to assess collected data.

**Results:**

Three hundred and fifty four senior dental students responded at a response rate of 88.5%. Thirty three percent respondents did not have any practical experience of IRR. Students showed a clear preference for CRR with regards to aesthetics (71.4%), passive fit (55.3%), fabrication ease (57.3%) and fracture resistance (40%). SRR were considered to provide better retention (59.6%), soft tissue health (51.1%) and ease of retrievability (72%). Nearly 40% of students agreed that they did not get sufficient information related to IRR in undergraduate courses.

**Conclusions:**

Clinical training of IRR is compromised in the undergraduate curriculum in dental schools of Saudi Arabia. The knowledge of dental students regarding IRR was broadly in line with current evidence.

## INTRODUCTION

Excellent survival rates of dental implant restorations have lead to their extensive use in the rehabilitation of patients through the years.^1^,^2^ Multiple factors influence the clinical success with dental implants, including oral hygiene, systemic patient health (such as diabetes and osteoporosis), patient habits (smoking), type of suprastructure (Cement or screw retained) and occlusal loads.^3^-^5^ The choice of implant restoration type [cement retained restorations (CRR) or screw retained restorations (SRR)] is a critical decision and is influenced by factors like mechanical complication, esthetic outcomes, ease of maintenance and financial implications. Both implant restoration types (CRR and SRR) have clinical and biological strengths and weaknesses. For instance, CRR offer better passivity of fit, aesthetics, occlusion and ease of fabrication.^6^,^7^ However, retention, retrieve ability and health of surrounding tissues are the rewards of utilizing SRR.^8^ A large number of implant complications are associated with IRR (such as, de-cementation, veneer fracture, screw loosening).^13^,^14^

Undergraduate (UG) teaching is vital in the successful management of IRR by general dental practitioners (GDP).^9^ Lim et al, showed that 84% of the responding U.S. dental schools required students to complete an implant dentistry course as part of their pre-doctoral training.^10^ In a similar study, it was shown that an average of 36 hours of theoretical and pre-clinical teaching were given to UG students. In addition, 70% of dental schools reported that students assist or treat patients with implant prosthetics.^11^ In a related investigation, dental students of King Abdulaziz University (Jeddah, Saudi Arabia), reported that majority of them (78.8%) did not receive enough lectures about dental implants and all of them did not have sufficient training in implant dentistry thus expresses the need for improvements in UG teaching related to implant dentistry.^12^

Dental students represent the future dental clinicians providing primary dental care, and therefore should be trained and educated adequately with regards to IRR. Furthermore, it is essential to investigate the level of student education and their exposure to implant restorations in order to ascertain their competency and to plan update in current student UG curriculum. Although implant dentistry is taught to UG students in most universities across Saudi Arabia, however to our knowledge from indexed literature there are no studies presenting the practice and understanding of UG students towards this. Therefore the aim of this study was to assess the practice and knowledge of IRR among senior dental students in the kingdom of Saudi Arabia.

## METHODS

The study population included senior dental students (fifth year and interns) among the dental schools in Saudi Arabia. We excluded the dental schools, which were relatively new (absence of senior students), schools where implant treatment is not undertaken and where interns were less than 10 in number (due to remote locations). Considering a minimum of 10% as our target population among senior dental students (fifth year and internship students) in Saudi Arabia from the year 2013 to date. By random selection, we included five dental schools [King Saud University (KSU), Qassim University (QU), Prince Salman bin Abdulaziz University (SAU), Dammam University (DU) and Taibah University (TU)]; this sample consisted of 400 senior students. The ethical committee of College of Dentistry Research Center, King Saud University approved the study protocol (Ref No.FR-0105).

A structured questionnaire in English language was used as an instrument for data collection. The administered questionnaire was divided into four sections. The first section enquired about participants; affiliation, gender, level/year in college and practice of IRR. The second section had nine questions enquiring, which implant restoration (CRR or SRR) better provides the properties desired in these restorations. These desired factors included esthetic outcome, cost effectiveness, ease of fabrication, expertise required for provision, retrievability, retention, passivity of fit, fracture resistance and surrounding tissue health. In the third part of the questionnaire (one question), participants were asked to grade factors considered important in selection of implant-retained prosthesis according to their clinical significance. The significance level scores ranged from one to five (0-1: very insignificant 1-2: insignificant 2-3: neutral 3-4: significant 4-5: Very significant). The last section (three questions) evaluated student’s perception of their knowledge and methods for further education in relation to the subject. This resulted in a total of seventeen questions.

Four hundred questionnaires along with a cover letter stating the instructions, rationale and purpose of the survey, were emailed and hand distributed among fifth year students and students in their internship in five dental schools (KSU, QU, SAU, DU and TU). Frequency distribution, average significance weightage (ASW) and statistical significance (p = 0.05) was assessed using descriptive analysis, analysis of variance (ANOVA) and student t-test. The data was coded, tabulated, and analyzed using Statistical package for social science (SPSS version 17).

## RESULTS

Three hundred fifty four students responded at a rate of 88.5% (male: 87.4, female: 91.50). All questionnaires were completely answered. The distribution of student affiliation is presented in Table-I. Of the total participants, 72.5% (n=257) were males and 27.4% (n=97) were females. 52.4% (n=207) were dental interns, however 41.4% (n=147) were fifth year students. Thirty three percent of respondents did not have any practical experience of implant restorations, whereas, 66% (males: 60.31%, females: 58.76%) had restored at least one dental implant. With regards to practical experience of IRR, male and female participants were statistically similar (p = 0.156) (Table-II).

**Table-I:** Affiliation of participating students.

Student Affiliation	Percentage of participants (N)
King Saud University	55 (194)
Qassim University	10 (35)
Prince Salman Bin Abdulaziz University	12 (44)
Dammam University	16 (56)
Taibah University	7 (27)

**Table-II:** Practical experience of implant retained restorations among participants.

		Percentage of restored implants (N)			P value
Dental School		None	1 to 5	> 5	
	KSU	18.36(65)	32.20(114)	4.23(15)	0.002*^†^
	QU	3.67 (13)	5.64 (20)	0.56 (2)	
	SAU	4.23 (15)	6.77 (24)	0.84 (3)	
	DU	4.80 (17)	9.88 (35)	1.12 (4)	
	TU	2.25 (8)	3.56 (19)	0.00 (0)	
Gender	Male	24.85 (88)	43.78 1(55)	3.95 (14)	0.156§
	Female	8.47(30)	16.10 (57)	2.82 (10)	
Academic level	Fifth yr Student	10.45 (37)	20.09 (103)	1.97 (7)	<0.001*^§^
	Interns	20. 88 (81)	30.79 (109)	4.80 (17)	

* Significant

† Analysis of variance(ANOVA)was performed

§ t-test was performed

Students showed a clear preference of CRR with regards to aesthetics (71.4%), passive fit (55.3%), fabrication ease (57.3%) and fracture resistance (40%). SRR were considered to provide better retention (59.6%), good soft tissue health (51.1%) and ease of retrievability (72%), however students also considered SRR to require higher technical expertise (42.6%). Regarding cost-effectiveness of implant restorations, 31% and 22% of students preferred SRR and CRR respectively (Table-III).

**Table-III:** Numerical summary of participant responses to survey questions.

No	Questions	Screw retained crown (%)	Cement retained crowns (%)	Both are same (%)	Don’t know (%)
1	Which restoration gives better aesthetics?	16.38	71.46	7.34	4.80
2	Which restoration is cost effective?	31.92	22.31	22.59	23.1
3	Which restoration is easier to fabricate?	25.14	57.34	6.77	10.73
4	Which restoration requires higher level of expertise?	42.65	21.18	21.18	14.97
5	Which restoration is easier to retrieve?	72.0	14.40	5.08	8.47
6	Which restoration has better retention?	59.60	27.96	9.32	3.1
7	Which restoration has better passivity of fit?	21.75	55.36	8.75	14.12
8	Which restoration has better fracture resistance?	27.68	39.83	9.60	22.88
9	Which restoration is more likely to disrupt surrounding tissue health?	23.1	51.1	8.75	16.94

The significance level of factors influencing selection between SRR and CRR is presented in Table-IV. Factors including aesthetics (4.23), soft tissue health (4.18) and retention (4.08) were considered the most important in decision making for IRR (average significance weightage [ASW] ≥ 4). The least important factors included cost-effectiveness (3.23), required expertise (3.45) and ease of fabrication (3.49).

**Table-IV:** Significance scores (percentage) for factors influencing selection of implant restorations.

Factors	Very Insignificant	Insignificant	Neutral	Significant	Very Significant	Average Weighted
Aesthetics	23 (6.49)	7 (4.80)	38 (10.73)	81 (22.88)	205 (58.75)	4.23
Soft tissue health	16 (4.51)	5 (1.41)	58 (16.38)	95 (26.83)	180 (50.84)	4.18
Retention	28 (7.90)	8 (2.25)	47 (13.27)	103 (29.09)	168 (47.45)	4.08
Fracture resistance	12 (3.38)	14 (3.95)	70 (19.77)	136 (38.41)	122 (34.46)	3.96
Passivity of fit	13 (3.67)	26 (7.34)	74 (20.90)	136 (38.41)	105 (29.66)	3.83
Ease of retrieval	19 (5.36)	33 (9.32)	108 (30.50)	97 (27.40)	97 (27.40)	3.62
Ease of Fabrication	23 (6.49)	49 (13.84)	102 (28.81)	91 (25.70)	89 (25.14)	3.49
Required Expertise	15 (4.23)	28 (7.90)	155 (43.78)	92 (25.98)	64 (18.07)	3.45
Cost-effectiveness	26(7.34)	103 (29.09)	92(25.98)	39(11.01)	96 (27.11)	3.23

Almost 40% of students agreed that they did not get sufficient information in their UG course and nearly 50% (n=174) of the students preferred to have more information. The favored method for better training and education related to IRR included, increasing clinical exposure in UG curriculum (46%, n=163), presence of structured dedicated courses in UG teaching (33%, n=117), short continued dental education programs and workshops (12.7%, n=45) and one-year modular courses (8.1%, n=29).

## DISCUSSION

This study presents data regarding practice and knowledge of dental implant restorations (SRR and CRR) among senior dental students (fifth year and interns) of Saudi Arabia. Fifth year dental students and interns were included in the study, as they are taught courses related to implant dentistry (such as prosthodontics, periodontology and oral surgery) in the final year of the dental curriculum and internship provides further clinical exposure aimed at training of implant-supported restorations of future GDP. The response rate for survey questionnaire was 88.5%. Similar response rates have been reported previously by Baruch and Brooks.^15^

An interesting finding in the present study was that 33% of the participants did not have any clinical exposure to IRR during their UG training. A possible explanation for this finding could be related to the absence of clinical competency requirement for implant prosthodontics for students in the comprehensive clinical dentistry courses. A report published by the American Dental Education Association (ADEA) regarding the education of implant dentistry in the dental schools in America, concluded that there were no fixed competency requirements in clinical courses for implant dentistry in the UG curriculum.^10^ As a result students can fulfill their minimum clinical case requirements with patients without the need of dental implants. It is recommended, that all aspects of implant dentistry should be an integral part of UG curriculum.^16^ Therefore, it is possible that if curriculums are revised in accordance with the current standards of dental education in Europe and America, an improvement in the practice of implant dentistry for dental students in Saudi Arabia is more likely.^10^,^17^

In the present study, senior dental students considered CRR to be superior to SRR with regards to aesthetics, passivity of fit, ease of fabrication and fracture resistance. These opinions appear to be in line with the established standards in implant dentistry.^8^,^18^ Where planned implant positioning is not possible, aesthetic outcome of SRR can be compromised by the presence of screw access hole.^8^ It is popular belief that CRR are more likely to achieve a passive fit,^18^,^19^ however, studies comparing CRR and SRR for passive fit have shown no difference.^20^ Moreover, SRR require extra components and expertise resulting in a comparatively costly and technique sensitive process. Furthermore, Presence of unsupported ceramic in SRR, result in increased incidence of fractures.^21^

In the outcomes of the present study, students considered SRR to offer better retention, soft tissue health and retrievability than CRR. It is known that, SRR allow for better peri-implant soft tissue attachment formation than CRR due to a cement free peri-implant environment.^8^ Moreover, SRR allow retrievability without much challenge and complications due to the presence of screw access hole.^22^ Furthermore, retention for SRR is considered more versatile due to the direct engagement of restorative screw on the implant.^8^,^23^

In the present study, nearly 40% respondents agreed that they did not get sufficient information on IRR, and almost 50% of the participants expressed the need for incorporation of further information regarding the subject in the UG curriculum. These findings emphasize the need for a revision of UG curriculum and reinforce the need for incorporation of structured courses dedicated to implant dentistry. Participants also expressed that there should be increased clinical exposure of implant restorations in the UG education (46%) and demanded structured implant dentistry courses in UG curriculum (33%). Students did not show much preference for short continued dental education implant workshops (12.7%). In a similar study, it has been reported that only 15% of interns preferred implant information updates from vendor led short workshops.^24^

It is reported that in most schools of America, there is a lack of adequately trained UG faculty members in subject of dental implants.^10^ This aspect although vital should be investigated, and is a limitation of the present study. Furthermore, attitude of GDP towards provision of implant restorations is directly related to their experience in providing the type of restoration.^25^ Therefore, the type of IRR (overdentures, SRR, CRR, crowns and FPDs) provided by senior dental students in their UG training is critical in understanding the abilities of GDP. However this was not assessed in the present study.

Therefore it is recommended that, education and training of UG students in the subject of dental implant restorations needs revision and structured dedicated courses on implant dentistry should be introduced to improve clinical exposure of students to this essential treatment modality. Moreover, UG clinical competency requirement for implant prosthodontics should be a part of UG implant courses outline.

## CONCLUSION

It is concluded that practical experience of IRR for senior dental students in UG training is compromised. In addition, 50% of senior dental students desired more information regarding implant restorations in the UG curriculum. Increased clinical exposure and incorporation of structured implant dentistry courses in UG curriculum are preferred by students to improve implant training.

## References

[1] Le M, Papia E, Larsson C (2015). The clinical success of tooth- and implant-supported zirconia-based fixed dental prostheses. A systematic review. J Oral Rehabil.

[2] Da Silva JD, Kazimiroff J, Papas A, Curro FA, Thompson VP, Vena DA (2014). Outcomes of implants and restorations placed in general dental practices: a retrospective study by the Practitioners Engaged in Applied Research and Learning (PEARL) Network. J Am Dent Assoc.

[3] Geckili O, Bilhan H, Geckili E, Cilingir A, Mumcu E, Bural C (2014). Evaluation of possible prognostic factors for the success, survival, and failure of dental implants. Implant Dent.

[4] Vohra F, Al-Rifaiy MQ, Almas K, Javed F (2014). Efficacy of systemic bisphosphonate delivery on osseointegration of implants under osteoporotic conditions: lessons from animal studies. Arch Oral Biol.

[5] Diz P, Scully C, Sanz M (2013). Dental implants in the medically compromised patient. J Dent.

[6] Hebel KS, Gajjar RC (1997). Cement-retained versus screw-retained implant restorations: achieving optimal occlusion and esthetics in implant dentistry. J Prosthet Dent.

[7] Misch CE (1995). Screw-retained versus cement-retained implant-supported prostheses. Pract Periodontics Aesthet Dent.

[8] Michalakis KX, Hirayama H, Garefis PD (2003). Cement-retained versus screw-retained implant restorations: a critical review. Int J Oral Maxillofac Implants.

[9] Mattheos N, Ivanovski S, Heitz-Mayfield L, Klineberg I, Sambrook P, Scholz S (2010). University teaching of implant dentistry: guidelines for education of dental undergraduate students and general dental practitioners. An Australian consensus document. Aust Dent J.

[10] Petropoulos VC, Arbree NS, Tarnow D, Rethman M, Malmquist J, Valachovic R (2006). Teaching implant dentistry in the predoctoral curriculum: a report from the ADEA Implant Workshop’s survey of deans. J Dent Educ.

[11] De Bruyn H, Koole S, Mattheos N, Lang NP (2009). A survey on undergraduate implant dentistry education in Europe. Eur J Dent Educ.

[12] Aljohani HA, Alghamdi AS (2009). Predoctoral dental implant education at King Abdulaziz University. Saudi Dent J.

[13] Vohra F, Al Fawaz A (2013). Repair of a fractured implant overdenture gold bar: A clinical and laboratory technique report. Eur J Dent.

[14] Jung RE, Zembic A, Pjetursson BE, Zwahlen M, Thoma DS (2012). Systematic review of the survival rate and the incidence of biological, technical, and aesthetic complications of single crowns on implants reported in longitudinal studies with a mean follow-up of 5 years. Clin Oral Implants Res.

[15] Baruch Y, Brooks H (2008). Survey response rate levels and trends in organizational research. Human Relations.

[16] Mattheos N, Albrektsson T, Buser D, De Bruyn H, Donos N, Hjorting Hansen E (2009). Teaching and assessment of implant dentistry in undergraduate and postgraduate education: a European consensus. Eur J Dent Educ.

[17] Lim MV, Afsharzand Z, Rashedi B, Petropoulos VC (2005). Predoctoral implant education in U.S. dental schools. J Prosthodont.

[18] Chee W, Felton DA, Johnson PF, Sullivan DY (1999). Cemented versus screw-retained implant prostheses: which is better?. Int J Oral Maxillofac Implants.

[19] Duyck J, Naert I (2002). Influence of prosthesis fit and the effect of a luting system on the prosthetic connection preload: an in vitro study. Int J Prosthodont.

[20] Heckmann SM, Karl M, Wichmann MG, Winter W, Graef F, Taylor TD (2004). Cement fixation and screw retention: parameters of passive fit. An in vitro study of three-unit implant-supported fixed partial dentures. Clin Oral Implants Res.

[21] Zarone F, Sorrentino R, Traini T, Di lorio D, Caputi S (2007). Fracture resistance of implant-supported screw- versus cement-retained porcelain fused to metal single crowns: SEM fractographic analysis. Dent Mater.

[22] Guichet DL, Caputo AA, Choi H, Sorensen JA (2000). Passivity of fit and marginal opening in screw- or cement-retained implant fixed partial denture designs. Int J Oral Maxillofac Implants.

[23] Breeding LC, Dixon DL, Bogacki MT, Tietge JD (1992). Use of luting agents with an implant system: Part I. J Prosthet Dent.

[24] Chaudhary S, Gowda TM, Kumar TA, Mehta DS (2013). Knowledge and attitudes of dental interns in Karnataka state, India, regarding implants. J Dent Educ.

[25] Lang-Hua BH, Lang NP, Lo EC, McGrath CP (2013). Attitudes of general dental practitioners towards implant dentistry in an environment with widespread provision of implant therapy. Clin Oral Implants Res.

